# Strongyloides Duodenitis in an Immunosuppressed Patient with Lupus Nephritis

**Published:** 2018-02-28

**Authors:** Ramprasad Jegadeesan, Tharani Sundararajan, Roshni Jain, Tejashree Karnik, Zalina Ardasenov, Elena Sidorenko

**Affiliations:** 1 Department of Internal Medicine, Division of Gastroenterology, Hepatology and Motility, University of Kansas School of Medicine-Kansas City; 2 Department of Psychiatry and Behavioral Sciences, University of Kansas School of Medicine-Kansas City; 3 Department of Pathology and Laboratory Medicine, University of Kansas School of Medicine-Kansas City; 4 Department of Internal Medicine, Division of General, Geriatric and Hospital Medicine, University of Kansas School of Medicine-Kansas City

**Keywords:** Strongyloides stercoralis, strongyloidiasis, immunocompromised patients, lupus nephritis, esophagogastroduodenoscopy

## Introduction

*Strongyloides stercoralis* is an intestinal nematode acquired in the tropics or subtropics.^1^–^3^ In an immunocompetent host, infection with *S stercoralis* produces a negligible or minimally symptomatic chronic disease. However, when immunosuppression impairs the host’s regulatory function, infection with *S stercoralis* can lead to hyperinfection syndrome and disseminated strongyloidiasis, which can be fatal. Diagnosing strongyloidiasis early is important because the case fatality rate of hyperinfection syndrome in immunosuppressed patients is between 50% and 86%.^4^–^5^ We report the case of a woman from Laos with recently diagnosed lupus nephritis who presented with complaints of abdominal pain, vomiting, and diarrhea.

## Case report

A 57-year-old Laotian female with a past medical history of systemic lupus erythematosus presented as a transfer from an outside facility for further management of a lupus nephritis flare. She was admitted at the outside facility five days before transfer due to complaints of shortness of breath on exertion, leg swelling, intermittent non-bloody watery diarrhea, nausea, vomiting, abdominal pain, bloating, and worsening proteinuria over two weeks. She had a history of lupus for the past few years. Due to worsening renal function, she had a renal biopsy three months prior to presentation which was consistent with lupus nephritis (stage V membranous nephropathy). She was placed on prednisone taper and mycophenolate mofetil two months prior at the outside facility. At presentation, the patient reported four days of melena but denied bloody stools. She did not report any recent travel outside the United States, any sick contacts, or recent antibiotic use.

On examination, the patient demonstrated pallor and alopecia. Cardiovascular and respiratory examinations were unremarkable, and her abdomen was soft without any tenderness or abdominal distension. Bilateral knee swelling and pedal edema were noted. Skin examination was normal. She had normal vital signs on admission at our hospital. Labs done on admission demonstrated normocytic anemia with hemoglobin of 9.8 gm/dl (reference range 12 – 15 gm/dl), leukocytosis with white blood cell count of 13.3 K/UL (reference range 4.5 – 11.0 K/UL, differential count not checked), elevated ESR of 41 mm/hr (reference range 0 – 30 mm/hr), elevated CRP of 1.64 mg/dl (reference range < 1.0 mg/dl), and normal renal and liver function tests. Urine culture was positive for *Escherichia Coli*, which was treated with appropriate antibiotics.

**Figure 1:**
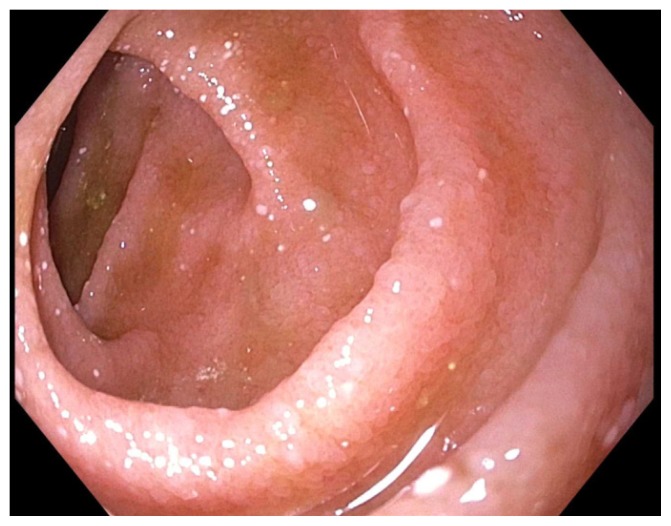
Normal endoscopic appearance of the second part of the duodenum.

**Figure 2:**
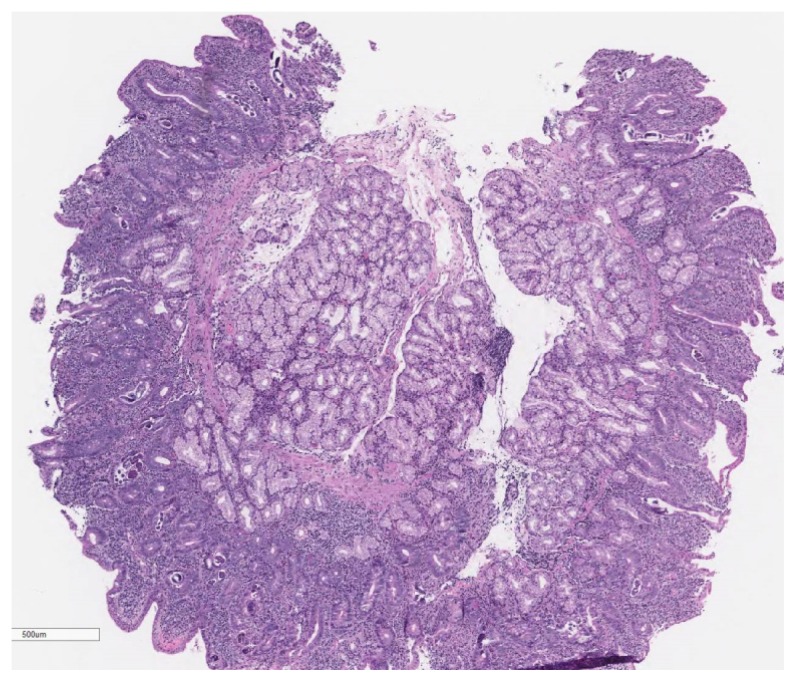
The duodenal biopsy showed a dense inflammatory infiltrate with eosinophils, lymphocytes, plasma cells, and few neutrophils involving and causing expansion of the lamina propria and shortening of the villi (hematoxylin and eosin stain, low power field image).

**Figure 3:**
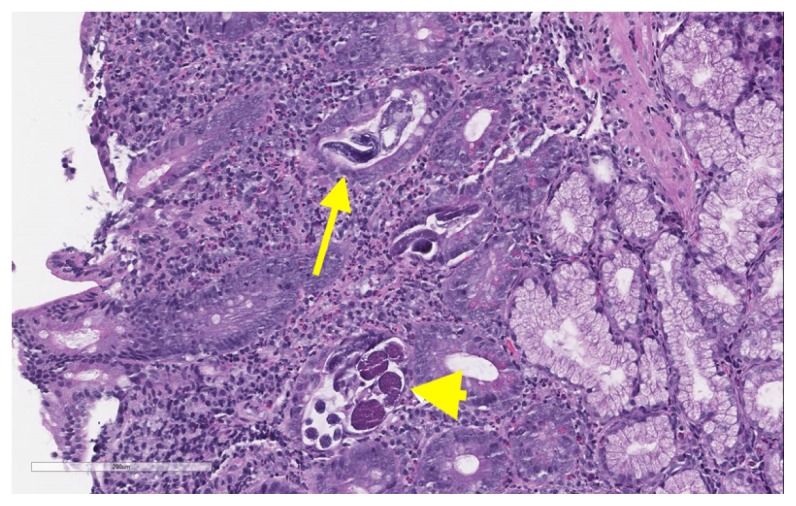
*Strongyloides stercoralis* is identified within the crypts of the duodenum. Rhabditiform and filariform larvae (yellow arrow) along with adult females with eggs (cross section; yellow arrow head) are seen (hematoxylin and eosin stain, high power field image).

**Figure 4:**
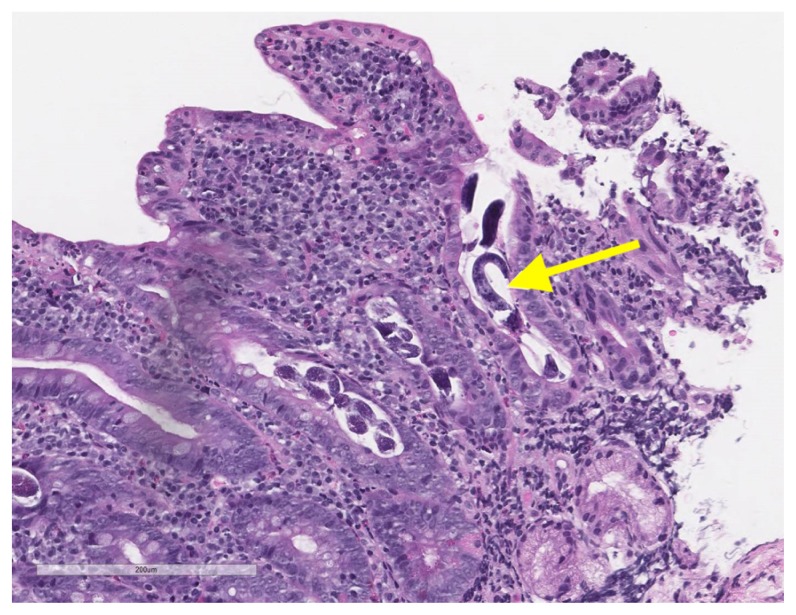
Longitudinal section of the adult *Strongyloides stercoralis* worm (yellow arrow) (hematoxylin and eosin stain, high power field image).

Due to melena and a two-gram drop in hemoglobin, upper and lower endoscopy was performed. Esophagogastroduodenoscopy (EGD) showed a large gastric submucosal mass with the appearance of cystic lesion in the cardia, which was not biopsied, and erythematous mucosa in the stomach and normal appearing duodenum (Figure 1), which was biopsied due to history of watery diarrhea. Duodenal biopsies showed marked duodenitis with parasitic organisms favoring duodenal strongyloidiasis (Figures 2, 3, and 4). Colonoscopy demonstrated a 4 mm cecal polyp, consistent with tubular adenoma, and 7 mm sigmoid colon polyp, which were removed. Colonoscopy also revealed normal ileum and colonic mucosa.

Following the diagnosis of duodenal strongyloidiasis by biopsy, the patient’s absolute eosinophil count was elevated at 1137/UL (reference range 0 – 450/UL). Strongyloides antibody (IgG) and Immunoglobin E (IgE) were positive. Stool examination was positive for *Strongyloides stercoralis*. Sputum examination was negative, as was the CT scan of the chest, providing no definite evidence of disseminated disease. We did not find the source of melena but hemoglobin stabilized during the hospital stay. The patient was treated with 9 mg ivermectin for seven days and finally discharged on 9 mg ivermectin monthly for six months.

Two weeks following discharge, the patient was readmitted with complaints of melena, fatigue, and abdominal pain. Stool examination was negative for *S stercoralis*, and peripheral eosinophilia had resolved. She was evaluated further with EGD for melena and noted to have gastric cardia submucosal mass and otherwise normal stomach. Duodenal biopsy obtained at that time did not show evidence of parasitic organisms.

## Discussion

Roughly 100 million people are infected by *Strongyloides stercoralis* in tropical and subtropical areas.^1^–^3^ It is the fourth most common nematode infection in the world and is prevalent in Africa, Asia, Southeast Asia, Central America, South America, and parts of the eastern United States. Humans acquire strongyloidiasis when larvae penetrate the skin and migrate to reach the duodenum and upper jejunum to mature. The rhabditiform larvae develop into filariform larvae within the intestines, which may penetrate the colonic wall or perianal skin and complete an internal cycle (auto-infection). This phenomenon of autoinfection is responsible for the persistence of infection virtually indefinitely in infected hosts.^6^–^7^

Chronic infection with *S stercoralis* is most often asymptomatic in an immunocompetent host.^8^,^9^ Hyperinfection syndrome manifesting as locally destructive bowel or lung disease and disseminated strongyloidiasis can occur in patients with impaired cell-mediated immunity (such as patients with transplant, patients receiving steroids or immunosuppressive therapy). Gastrointestinal and pulmonary symptoms are common but non-specific, and include abdominal pain, diarrhea, vomiting, adynamic ileus, small bowel obstruction (SBO) and protein-losing enteropathy, as well as pneumonia.^10^,^11^ Endoscopic findings of the duodenum in strongyloidiasis as reported by several prior case reports, includes normal mucosa, edema, erythema, erosion, swollen folds, fine granule, tiny ulcer, polyps, hemorrhage, megaduodenum, deformity, and stenosis.^12^

Definitive diagnosis of strongyloidiasis usually is made by detecting larvae in the stool, sputum, or tissue biopsy. A single stool examination is unable to detect larvae in up to 70% of cases, but repeat stool examinations have been shown to improve diagnostic sensitivity.^13^,^14^ Due to the need for multiple stool samples to detect *S stercoralis* larvae, it is important to recognize that not identifying larvae in the stool does not imply an absence of infection. Upper and lower endoscopy also can establish the diagnosis of strongyloidiasis, as larvae may be seen on biopsies of the affected mucosa as noted in our patient.^15^ Thompson et al.^16^ showed that a minimum of six biopsies obtained from duodenal lesions resulted in a 100% histopathologic yield in diagnosing duodenal strongyloidiasis. Larvae can be demonstrated on normal appearing duodenal mucosa as shown in our patients and the endoscopist should consider biopsy of normal appearing duodenum if there is clinical suspicion of Strongyloides infection.

Ivermectin is the drug of choice as per World Health Organization.^13^ In disseminated disease, hyperinfection syndrome, and the immunocompromised patients, ivermectin is given daily until symptoms cease and stool samples are negative for *S stercoralis* larvae for at least two weeks (1 autoinfection cycle). Diagnosing strongyloidiasis early is important, as almost all deaths due to helminths in the United States are due to *S stercoralis* hyperinfection.^17^ It appears that our patient, an immigrant from an endemic area, was a chronic carrier of *S stercoralis* and the recent treatment with steroids precipitated hyperinfection syndrome leading to marked duodenitis. Timely diagnosis and providing therapy without delay in our patient most likely decreased her mortality as case fatality rate of hyperinfection syndrome in patients with diminished cellular immunity is between 50% and 86%.^4^,^5^

In summary, we presented a case of hyperinfection syndrome with *Strongyloides stercoralis* with improved clinical outcome due to early intervention and therapy. Clinicians must have a high suspicion of *S stercoralis* infection, especially in immunocompromised patients, as strongyloidiasis is difficult to diagnose and delaying therapy can have fatal consequences.
